# Using Pan RNA-Seq Analysis to Reveal the Ubiquitous Existence of 5′ and 3′ End Small RNAs

**DOI:** 10.3389/fgene.2019.00105

**Published:** 2019-02-14

**Authors:** Xiaofeng Xu, Haishuo Ji, Xiufeng Jin, Zhi Cheng, Xue Yao, Yanqiang Liu, Qiang Zhao, Tao Zhang, Jishou Ruan, Wenjun Bu, Ze Chen, Shan Gao

**Affiliations:** ^1^State Key Laboratory of Veterinary Etiological Biology, Key Laboratory of Veterinary Parasitology of Gansu Province, Lanzhou Veterinary Research Institute, Chinese Academy of Agricultural Sciences, Lanzhou, China; ^2^College of Life Sciences, Nankai University, Tianjin, China; ^3^Institute of Statistics, Nankai University, Tianjin, China; ^4^Department of Orthopedics, Tianjin Medical University General Hospital, Tianjin, China; ^5^School of Mathematical Sciences, Nankai University, Tianjin, China

**Keywords:** small RNA, 5′ end, 3′ end, Pan RNA-seq, genome annotation

## Abstract

In this study, we used pan RNA-seq analysis to reveal the ubiquitous existence of both 5′ and 3′ end small RNAs (5′ and 3′ sRNAs). 5′ and 3′ sRNAs alone can be used to annotate nuclear non-coding and mitochondrial genes at 1-bp resolution and identify new steady RNAs, which are usually transcribed from functional genes. Then, we provided a simple and cost effective way for the annotation of nuclear non-coding and mitochondrial genes and the identification of new steady RNAs, particularly long non-coding RNAs (lncRNAs). Using 5′ and 3′ sRNAs, the annotation of human mitochondrial was corrected and a novel ncRNA named non-coding mitochondrial RNA 1 (ncMT1) was reported for the first time in this study. We also found that most of human tRNA genes have downstream lncRNA genes as lncTRS-TGA1-1 and corrected the misunderstanding of them in previous studies. Using 5′, 3′, and intronic sRNAs, we reported for the first time that enzymatic double-stranded RNA (dsRNA) cleavage and RNA interference (RNAi) might be involved in the RNA degradation and gene expression regulation of U1 snRNA in human. We provided a different perspective on the regulation of gene expression in U1 snRNA. We also provided a novel view on cancer and virus-induced diseases, leading to find diagnostics or therapy targets from the ribonuclease III (RNase III) family and its related pathways. Our findings pave the way toward a rediscovery of dsRNA cleavage and RNAi, challenging classical theories.

## Introduction

RNA sequencing (RNA-seq), performed primarily on next-generation sequencing (NGS) platforms, is widely used to measure the expression levels of multiple genes simultaneously, with higher accuracy than Serial Analysis of Gene Expression (SAGE) and microarray (Gao et al., 2014). RNA-seq is also used for genome annotation, enabling the study of gene transcription, RNA processing and various other biological functions. In particular, RNA-seq or small RNA sequencing (sRNA-seq) is indispensable for the annotation of non-coding genes, while the annotation of protein-coding genes can be conducted based on the analysis of protein codons. However, RNA-seq cannot be used to obtain full-length transcripts by *de novo* assembly or alignment. Both PacBio full-length transcripts (PacBio cDNA-seq) (Ren et al., 2016) and Nanopore cDNA sequencing (Nanopore cDNA-seq) (Gao et al., 2014) can be used to obtain full-length transcripts of mature RNAs or RNA precursors (Gao et al., 2016). PacBio cDNA-seq produces reads with lower error rates than Nanopore cDNA-seq, while Nanopore cDNA-seq can produce longer reads than PacBio cDNA-seq. However, neither PacBio cDNA-seq nor Nanopore cDNA-seq can provide the exact 3′-end information of transcripts (e.g., polyA regions) due to reverse transcription. This results from the fact that primers anneal to random positions located in the polyA or A-enriched regions within the transcripts to start reverse transcription. Nanopore direct RNA sequencing (Nanopore RNA-seq), as the only available sequencing technology which can sequence RNA directly (Garalde et al., 2018), theoretically can be used to obtain the full-length 3′ ends of transcripts. However, it cannot be used to obtain the exact 3′-end information of transcripts either, due to the high error rate of Nanopore RNA-seq data. Combined with specific capture or enrichment technologies, several other RNA-seq methods have been developed to extend the use of standard RNA-seq. Parallel Analysis of RNA Ends and sequencing (PARE-seq), Cap Analysis of Gene Expression and sequencing (CAGE-seq) and Precision nuclear Run-On and sequencing (PRO-seq) have been developed to identify the 5′ ends of mature RNAs (Bouvy-Liivrand et al., 2017). Polyadenylation sequencing (PA-seq) has been developed to identify the 3′ ends of mature RNAs (Ni et al., 2013). Global Run-On and sequencing (GRO-seq) has been developed to sequence nascent RNAs (Bouvy-Liivrand et al., 2017), which helps to determine the primary transcripts of genes.

In our previous studies, we used standard RNA-seq, sRNA-seq, PARE-seq, CAGE-seq, PRO-seq, PA-seq, GRO-seq, PacBio cDNA-seq, Nanopore cDNA-seq, and Nanopore RNA-seq etc to improve gene annotation, defined as pan RNA-seq analysis. Using pan RNA-seq analysis, we reported the corrected annotation of tick and human rRNA genes (Chen et al., 2017), insect mitochondrial genes (Gao et al., 2016) and human mitochondrial genes (Gao et al., 2017). We also reported two novel long non-coding RNAs (lncRNAs) found in human mitochondrial DNA (Gao et al., 2017). In addition, we unexpectedly detected the existence of 5′ and 3′ end small RNAs (5′ and 3′ sRNAs) in animal rRNA genes (Chen et al., 2017) and later proved the ubiquitous existence of 5′ and 3′ sRNAs in nuclear non-coding and mitochondrial genes. In this study, we demonstrated that 5′ and 3′ sRNAs alone can be used to annotate nuclear non-coding and mitochondrial genes at 1-bp resolution and identify new steady RNAs. Using public sRNA-seq data from the same species, this method provides a simple and cost-effective way to annotate nuclear non-coding and mitochondrial genes and identify new steady RNAs, which are defined to be against transient RNAs. Furthermore, 5′, 3′, and intronic sRNAs can be used to investigate RNA processing, maturation, degradation and even gene expression regulation. Using 5′, 3′, and intronic sRNAs, we revealed that enzymatic double-stranded RNA (dsRNA) cleavage initiates RNA interference (RNAi), which might be involved in the RNA degradation and gene expression regulation of U1 snRNA in human. Our findings pave the way toward a rediscovery of dsRNA cleavage and RNAi, challenging classical theories.

## Results

### Discovery of 5′ and 3′ sRNAs

**FIGURE 1:**
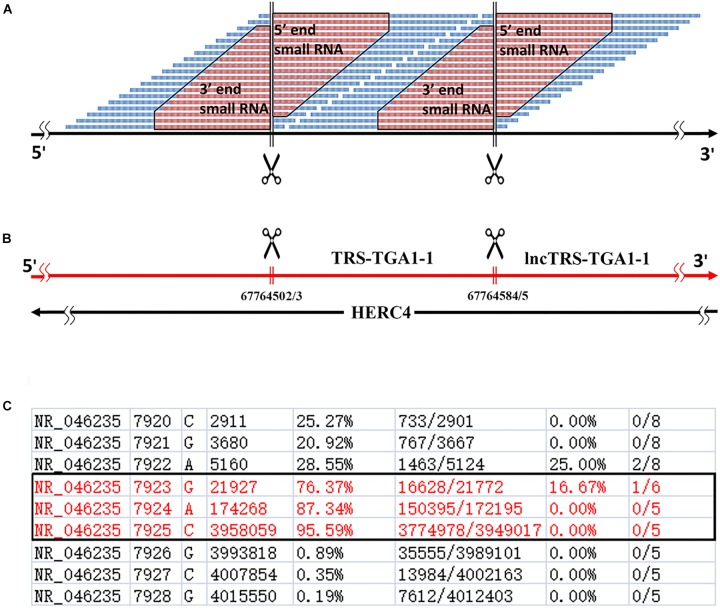
Definition of 5′ and 3′ sRNAs. **(A)** 5′ and 3′ sRNAs (in red color) are defined as sRNA-seq reads with lengths of 15∼50 bp, which are precisely aligned to the 5′ and 3′ ends of mature RNAs, respectively. The lengths of them vary progressively with 1-bp differences. This figure shows 5′ and 3′ sRNAs from a typical tRNA (in blue color). As for longer RNAs (e.g., snRNAs or rRNAs), there could be abundant sRNAs in the body. **(B)** 5-end format is defined to easily identify 5′ ends of mature RNAs using sRNA-seq data. **(C)** Human rRNA genes (RefSeq: NR_046235.1) were annotated using alignment results in the 5-end format. Among positions 7923, 7924, and 7925 with ratio1s (the 5th column) above 70%, the position 7925 with the highest ratio1 was determined as the 5′ end of 28S rRNA.

A genome-alignment map of sRNA data usually exhibits certain peaks or hotspots (Li et al., 2012) where the depths of these positions are much higher than those of other positions in the genome. In our previous study of human rRNA genes (Chen et al., 2017), we found that some peaks represented 5′ and 3′ sRNAs that existed ubiquitously in nuclear non-coding and mitochondrial genes in eukaryotes. Given that current sRNA-seq technologies usually provide sequences with short lengths, 5′ and 3′ sRNAs are defined as sRNA-seq reads with lengths of 15∼50 bp, which are precisely aligned to the 5′ and 3′ ends of mature RNAs, respectively (Figure 1A,B). They exhibit the following features: (1) 5′ and 3′ sRNAs are degraded fragments of mature RNAs and their lengths vary progressively with 1-bp differences; (2) the cleavage sites between 3′ sRNAs and their downstream 5′ sRNAs are not limited to one, but instead consist usually of three sites (Figure 1C), due to inexact cleavage by enzymes; and (3) 5′ and 3′ sRNAs of steady RNAs (e.g., 18S, 5.8S, and 28S rRNA) are significantly more abundant than their intronic sRNAs, while 5′ and 3′ sRNAs of transient RNAs (e.g., internal transcribed spacers of rRNA, ITS1, and ITS2) are not. The last criterion can be used to identify new steady RNAs, which are usually transcribed from functional genes. One example of a new steady RNA lncTRS-TGA1-1 and another example of two novel mitochondrial lncRNAs (MDL1 and MDL1AS) are introduced in the following paragraphs. In addition, we demonstrated that MDL1 and MDL1AS are two steady lncRNAs in human mitochondrial DNA with biological functions (Gao et al., 2017).

We used 5′ and 3′ sRNAs from one sRNA-seq dataset to annotate genes and used one CAGE-seq dataset, one GRO-seq dataset and one PacBio cDNA-seq dataset (section “Materials and Methods”) to validate the annotations. Later, we developed a simplified procedure for gene-annotation. Using only 5′ sRNAs, gene annotation can be reduced to the identification of the 5′ ends of mature RNAs. In doing so, the 3′ ends of their upstream mature RNAs and their cleavage sites can be derived (Figure 1A). We have defined a new file format, named “5-end format,” to easily identify the 5′ ends of mature RNAs. The new format is derived from the Pileup format (see section “Materials and Methods”) to include eight columns (Figure 1C) for each line providing information for a genomic position: (1) chromosome ID; (2) 1-based coordinate of this position; (3) reference base; (4) depth (the number of reads covering the position); (5) ratio-1 (the number of positive-stranded reads starting at this position divided by the total number of positive-stranded reads); (6) the number of positive-stranded reads starting at this position and the total number of positive-stranded reads; (7) ratio-2 (the number of negative-stranded reads starting at this position divided by the total number of negative-stranded reads); and (8) the number of negative-stranded reads starting at this position and the total number of negative-stranded reads. As the inexact cleavage in RNA processing results in two or three neighboring sites, we select the most occurred one for annotation. Using the 5-end format, the 5′ end of one mature RNA can easily be identified from two to three candidates (Figure 1C), the ratio-1s or ratio-2s of which must be above a threshold (e.g., 75%) and significantly higher than those of the positions surrounding them.

### 5′ and 3′ sRNAs in Nuclear Non-coding Genes

**FIGURE 2:**
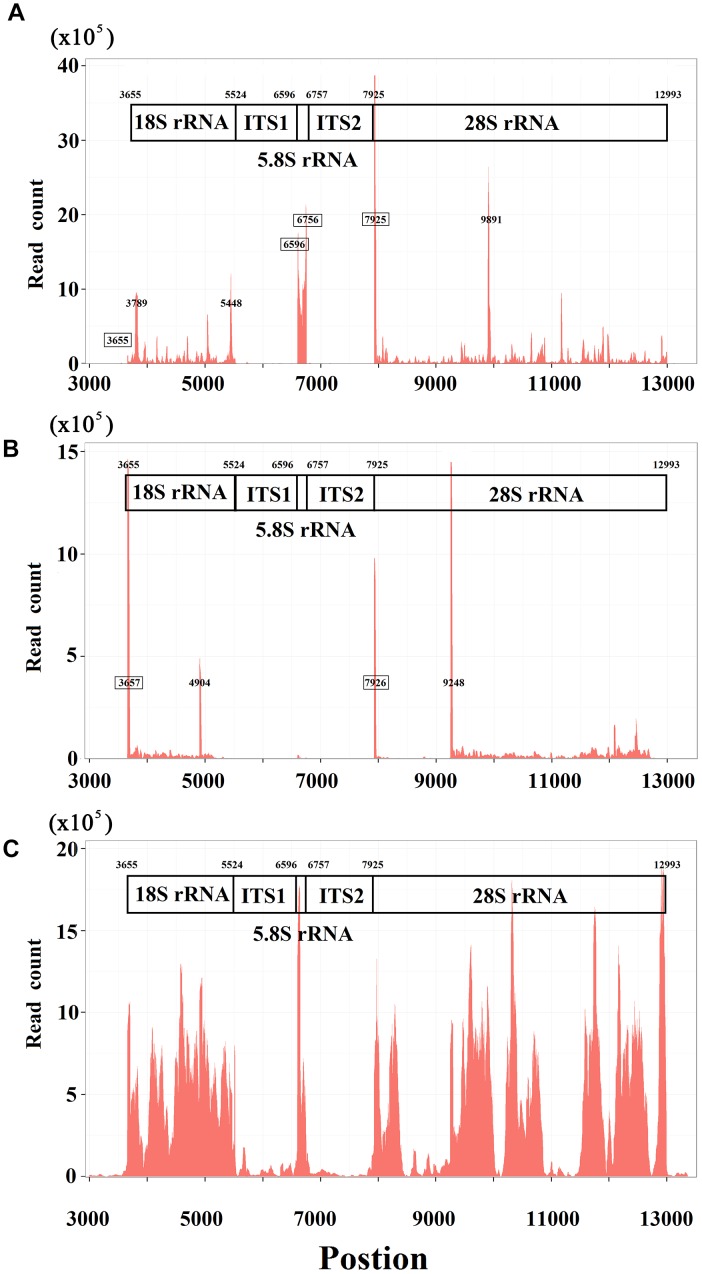
Genome-alignment maps using sRNA-seq, Cage-seq, and GRO-seq. This figure shows the count distribution of all aligned reads on the reference rRNA sequence (RefSeq: NR_046235.1). These reads are from one sRNA-seq dataset **(A)** one CAGE-seq dataset **(B)** and one GRO-seq dataset **(C)** the description of these datasets can be seen in the Section “Materials and Methods.” The identified 5′ and 3′ ends of mature RNAs are marked by boxes.

Using 5′ and 3′ sRNAs, we corrected the annotation of human rRNA genes. For the 5′ end of each mature RNA, we obtained two or three candidates and selected the position with the highest ratio-1 or ratio-2 to annotate genes on the positive or negative strands. For example, we obtained three positions, 7,923, 7,924, and 7,925, to identify the 5′ end of 28S rRNA and selected 7,925 for annotation (Figure 1C). In the same way, the 5′ ends of 18S and 5.8S rRNA were also identified using 5′ sRNAs. Then the 3′ ends of 18S, 5.8S, and 28S rRNA were identified using 3′ sRNAs. Finally, the annotations of ITS1 and ITS2 were derived using the annotations of 18S, 5.8S and 28S rRNA (Figure 2A). The corrected annotations of human rRNA genes (Table 1) were validated using the CAGE-seq dataset and the GRO-seq dataset (Figure 2B,C). Although the depth of 1,471,247 reads at position 6,601 was much higher than the depth of 647,406 reads at position 6,596 in the sRNA-seq dataset, the 5′ end of 5.8S rRNA annotated at position 6,601 with a ratio-1 of 35.42% (520,006/1,468,024) was still corrected as position 6,596 with a ratio-1 of 88.11% (569,882/646,805). In addition, the genome-alignment map using the sRNA-seq dataset showed that human rRNA genes had peaks at positions 6,596, 7,925, and 6,756 corresponding to the 5′ ends of 5.8S and 28S rRNA and the 3′ end of 5.8S rRNA, respectively (Figure 2A). The genome-alignment map using the CAGE-seq dataset showed that human rRNA genes had peaks at positions 3,657 and 7,926 corresponding to the 5′ ends of 18S and 28S rRNA, respectively (Figure 2B). This suggested that 18S and 28S rRNA could be capped by 5′ m^7^G or other caps, but 5.8S rRNA could at most be capped at a low level, if at all. By analyzing the 3′ sRNAs, we confirmed that mature rRNAs did not contain 3′ polyAs.

**Table 1:** Annotation of human rRNA genes with corrections.

Gene	Start	End	Start^∗^	End^∗^	Length^∗^
18S rRNA	3,655	5,523	3,655	5,523	1,869
ITS1	5,524	6,600	5,524	6,595^∗^	1,072
5.8S rRNA	6,601	6,757	6,596^∗^	6,756^∗^	161
ITS2	6,758	7,924	6,757^∗^	7,924	1,168
28S rRNA	7,925	12,994	7,925	12,993^∗^	5,069


*Human rRNA genes (RefSeq: NR_046235.1) were annotated using 5′ and 3′ sRNAs and ^∗^represented the corrected annotation.*

Lee et al. (2009) a novel class of sRNAs named tRNA-derived RNA fragments (tRFs) was introduced and three series of tRFs (tRF-5, tRF-3, and tRF-1) were identified using the sRNA-seq data of the human prostate cancer cell line by 454 deep sequencing. However, these authors did not achieve a full understanding of tRFs due to technological limitations and their small dataset size. Using pan RNA-seq analysis, we elucidated that the tRF-5 and tRF-3 series were 5′ and 3′ sRNAs from mature tRNAs and that the tRF-1 series were 5′ sRNAs from mature RNAs of the downstream genes (Figure 1B). As these 3′ sRNAs contained detailed 3′-end information of mature RNAs, we were able to assess factors related to tRNA processing, maturation and degradation by analyzing 12 mature tRNAs and their 42 precursors (Supplementary Table S1). For example, we found that there are four types of 3′ sRNAs derived from tRNAs: non-tail, C-, CC-, and CCA-tailed. The proportions of these four types were 5.26% (22,906/435,595), 12.36% (53,845/435,595), 13.81% (60,176/435,595), and 68.57% (298,668/435,595). In addition, we obtained the sequences of full-length mature tRNAs of all four types: with non-tail, C-, CC-, and CCA-tailed. Among these full-length mature tRNAs, 8,539 TRD-GTC2-1 tRNAs (for Asp) and 16,900 TRE-CTC1-1 tRNAs (for Glu) were obtained. These results suggested that 3′ sRNAs were produced by tRNA degradation during its synthesis, when CCAs were post-transcriptionally added to the 3′ ends of tRNAs one nucleotide at a time. Another example was the correction of TRL-TAG3-1’s annotation. Mature TRL-TAG3-1 (chr16:22195711-22195792) was annotated as an 82-nt sequence from the human genome with its 3′ cleavage site ACCGCTGCCA| cacctcagaa. Using 5′ and 3′ sRNAs, the 3′ cleavage site of TRL-TAG3-1 (chr16:22195710-22195792) was determined to be ACCGCTGCCAC| acctcagaa. The genome-alignment results using the CAGE-seq dataset showed that 5′ m^7^G or other caps of tRNAs did not exist. By analyzing the 3′ sRNAs, we confirmed that mature tRNAs did not contain 3′ polyAs. 5′ and 3′ sRNAs from all of the 13 mature tRNAs were represented by peaks in the genome-alignment maps, while only a few 3′ sRNAs of their upstream genes or 5′ sRNAs of their downstream genes were represented by peaks. Among the peaks from these upstream or downstream genes, the highest one was downstream of TRS-TGA1-1 (chr10:67764503-67764584), which suggested that this peak was the 5′ end of a new steady RNA which might be transcribed from a functional gene that had not been annotated in the current genome (version GRCh38/hg38). Since this new gene was downstream of TRS-TGA1-1, it was named by lncTRS-TGA1-1 (Figure 1B).

Small nuclear RNAs (snRNAs) include a class of small RNA molecules that are found within the splicing speckles and Cajal bodies of the cell nucleus in eukaryotic cells (Matera et al., 2007). snRNAs are always associated with a set of specific proteins and the complexes are referred to as small nuclear ribonucleoproteins (snRNPs). SnRNAs are also commonly referred to as U-RNAs and one well-known member is U1 snRNA (Cheng et al., 2017b). Using 5′ sRNAs, we confirmed annotations of U1, U2, U3, U4, U5, U6, and U7 (Supplementary Table S1). The genome-alignment results using the CAGE-seq dataset showed that U1, U2, U3, and U4 snRNAs could be capped by 5′ m^7^G or other caps, but U5, U6, and U7 snRNAs could at most be capped at a low level, if at all. By analyzing 3′ sRNAs, we confirmed that mature snRNAs did not contain 3′ polyAs. In addition, we did not find any new steady RNA upstream or downstream of seven snRNA genes.

### 5′ and 3′ sRNAs in Mitochondrial Genes

Using pan RNA-seq analysis, we confirmed that nuclear mitochondrial DNA segments (NUMTs) in the human genome did not transcribe into RNAs (Gao et al., 2017). This finding simplified the analysis of mitochondrial genes (e.g., mutation detection or quantification) using transcriptome data. In our previous study, we annotated two primary transcripts and 30 mature transcripts (tRNA^Ile^, tRNA^Gln^AS, tRNA^Met^, ND2, tRNA^Trp^, tRNA^Ala^AS/tRNA^Asn^AS/tRNA^Cys^AS/tRNA^Tyr^AS, COI, tRNA^Ser^AS, tRNA^Asp^, COII, tRNA^Lys^, ATP8/6, COIII, tRNA^Gly^, ND3, tRNA^Arg^, ND4L/4, tRNA^His^, tRNA^Ser^, tRNA^Leu^, ND5/ND6AS/tRNA^Glu^AS, Cytb, tRNA^Thr^, MDL1, tRNA^Phe^, 12S rRNA, tRNA^V al^, 16S rRNA, tRNA^Leu^, and ND1) on the H-strand at 1-bp resolution (Gao et al., 2017). We classified mitochondrial genes into tRNA, mRNA, rRNA, antisense tRNA (e.g., tRNA^Ser^AS), antisense mRNA (e.g., ND6AS), antisense rRNA and lncRNAs (e.g., MDL1 and MDL1AS) (Gao et al., 2017). Among the mature transcripts in human mitochondrial DNA, tRNA transcripts were tailed by 3′ CCAs, while other mature transcripts were tailed by 3′ polyAs. The analysis of 3′ sRNAs using the human931 sRNA-seq dataset (section “Materials and Methods”) showed that the maximum lengths of the polyAs in tRNA^Gln^AS, ND2, tRNA^Ala^AS-tRNA^Tyr^AS, COI, tRNA^Ser^AS, COII, ATP8/6, COIII, ND3, ND4L/4, ND5/ND6AS/tRNA^Glu^AS, Cytb, MDL1, 12S rRNA, 16S rRNA, and ND1 are 22, 13, 22, 17, 22, 24, 35, 22, 19, 22, 29, 25, 28, 32, 24, and 24, respectively. There was no significant difference in length distribution between polyAs in mRNAs and rRNAs, which updated the previous finding that the lengths of polyA tails in rRNAs could only be estimated within 3–4 or 6–7 bp (Stewart and Beckenbach, 2009). 3′ sRNAs containing polyAs or CCAs of different lengths were captured to demonstrate that 3′ sRNAs were produced by RNA degradation during its synthesis, when polyAs or CCAs were post-transcriptionally added to the 3′ ends of RNAs one nucleotide at a time. In this study, we also confirmed that mitochondrial mRNAs and rRNAs were capped by 5′ m^7^G or other caps (Gao et al., 2016). Our data also showed that MDL1AS, ND5/ND6AS/tRNA^Glu^AS and tRNA^Ala^AS/tRNA^Asn^AS/tRNA^Cys^AS/tRNA^Tyr^AS could be capped by 5′ m^7^G or other caps, but tRNA^Gln^AS and MDL1 could at most be capped at a low level, if at all. Although MDL1 was not capped by 5′ m^7^G or other caps as was MDL1AS, we still proposed that both MDL1 and MDL1AS were steady RNAs with biological functions, due to the fact that 5′ and 3′ sRNAs of MDL1 and MDL1AS were significantly more abundant than their intronic sRNAs. Further study showed that qPCR of MDL1 provided higher sensitivities than that of BAX/BCL2 and CASP3 in the detection of cell apoptosis (Liu C. et al., 2018).

**FIGURE 3:**
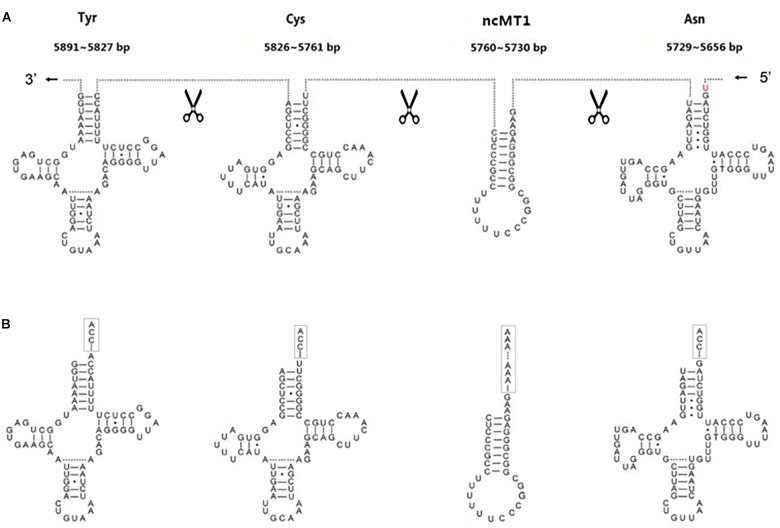
Corrected annotations of mitochondrial tRNAs. tRNA^Tyr^/tRNA^Cys^/tRNA^Asn^/tRNA^Ala^ is encoded by L-strand of human mitochondrial genome. tRNA^Ala^ is not showed, as it does not need correction. ncMT1 was first discovered in this study. **(A)** Mitochondrial tRNAs are annotated between two cleavage sites using the sRNA-seq based method, while they are annotated between two trimming sites and several nucleic acids in red color are missed using other existing methods. **(B)** The acceptor stem of a tRNA precursor is trimmed to contain a 1-bp overhang at the 3′ end. CCAs are post-transcriptionally added to the 3′ ends of tRNAs, one nucleotide at a time. A polyA tail is post-transcriptionally added to the 3′ end of ncMT1.

The annotation resolution of mitochondrial tRNAs is limited due to the complexity of tRNA processing. The annotation of consecutive tRNAs (e.g., tRNA^Tyr^/tRNA^Cys^/tRNA^Asn^/tRNA^Ala^ in human) is still difficult to solve (Figure 3). Using 5′ and 3′ sRNAs, we annotated the mitochondrial tRNAs of human at 1 bp resolution, which corrected the previous annotations (GenBank: NC_012920.1). Based on these results, we propose a mitochondrial tRNA processing model. One mitochondrial tRNA is cleaved from a mitochondrial primary transcript into a precursor (Figure 3A), and then the acceptor stem of the precursor is adenylated (e.g., tRNA^Tyr^ in human) or trimmed (e.g., tRNA^Asn^ in human) to contain a 1-bp overhang at the 3′ end. Finally, CCAs (for most of tRNAs) or CAs (e.g., tRNA^His^ in *Erthesina fullo*) are post-transcriptionally added to the 3′ ends of tRNAs, one nucleotide at a time. Using other existing methods, mitochondrial tRNAs are annotated between two trimming sites of their mature RNAs, which misses the information of the cleavage sites. Using our method, mitochondrial tRNAs are annotated between two cleavage sites and the information of the trimming sites (Figure 3B) can be derived using the mitochondrial tRNA processing model. As the new annotations cover both entire strands of mitochondrial genomes without any gaps or overlaps between neighboring genes, a novel ncRNA named non-coding mitochondrial RNA 1 (ncMT1) was first discovered between tRNA^Cys^ and tRNA^Asn^. ncMT1 (NC_012920.1:5730-5760) with a length of 31 nt is encoded by the L-strand and was identified as a steady RNA (Figure 3B). The mature ncMT1 has a polyA tail as mitochondrial mRNAs and rRNAs.

Mitochondrial genome annotation can also be confirmed by the “mitochondrial cleavage” model that we proposed in our previous study (Gao et al., 2017). The model is based on the fact that RNA cleavage is processed: (1) at 5′ and 3′ ends of tRNAs, (2) between mRNAs and mRNAs (e.g., ATP8/6 and COIII) except fusion gene [e.g., ATP8/6/COIII in *Platysternon megacephalum* (Liu J. et al., 2018)], (3) between antisense tRNAs and mRNAs (e.g., tRNA^Tyr^AS and COI) and (4) between mRNAs and antisense tRNAs (e.g., COI and tRNA^Ser^AS); but is not processed: (1) between mRNAs and antisense mRNAs (e.g., ND5 and ND6AS) or (2) between antisense RNAs (e.g., ND6AS and tRNA^Glu^AS or tRNA^Ala^AS/tRNA^Asn^AS/tRNA^Cys^AS/tRNA^Tyr^AS). This model does not rule out the possibility of a few cleavage events in tRNA^Ala^AS/tRNA^Asn^AS/tRNA^Cys^AS/tRNA^Tyr^AS, ND5/ND6AS/tRNA^Glu^AS or MDL1 (tRNA^Pro^AS/D-loop), however, such events are not necessary for their biological functions. Among these 30 mature transcripts on the H-strand, the enzymatic cleavage of COI/tRNA^Ser^AS was the most complicated in that the cleavage site contained an A-enriched region TCTAGACAAAAAA. The analysis of full-length transcripts using the PacBio cDNA-seq dataset (section “Materials and Methods”) showed that 95.65% (23,000/24,045) of COI/tRNA^Ser^AS was not further cleaved, while only 0.19% (45/24,045) and 4.16% (1,000/24,045) were cleaved at TCT| agacaaaaaa and TCTAGAC| aaaaaa, respectively. This suggested that COI/tRNA^Ser^AS was used as the template for the synthesis of proteins as ND5/ND6AS/tRNA^Glu^AS was used as the template. This model was used to correct annotations of non-coding RNAs in human mitochondrial DNA. For example, the identification of ND5/ND6AS/tRNA^Glu^AS, MDL1 and MDL1AS demonstrated that all other reported mitochondrial lncRNAs (Hedberg et al., 2018) could be degraded fragments of transient RNAs or random breaks during experimental processing. Another example included the observation that tRNA^Ala^AS-tRNA^Tyr^AS (NC_012920: 1318-1638) was not further cleaved for specific functions, which contradicted the hypothesis of a previous study (Seligmann, 2010).

We had previously determined that the first transcription initiation site (TIS) of the H-strand (IT_H1_) and the TIS of the L-strand (IT_L_) were at positions 561 and 407 of the human mitochondrial genome (RefSeq: NC_012920.1); however, the second TIS of the H-strand (IT_H2_) was not determined using only sRNA-seq data (Gao et al., 2017). By the analysis using sRNA-seq and GRO-seq data, IT_H2_ was determined to be at position 647 or 648 that was also the 5′ end of 12S rRNA. This finding went against the long-standing claim that IT_H2_ was at position 638 (Montoya and Attardi, 1982). Using pan RNA-seq analysis, we found that all of the TISs (IT_H1,_ IT_H2_ and IT_L_) could be capped by 5′ m^7^G or other caps. We also found polyAs before the TISs, particularly GAG_6_A_0∼11_ before IT_H1_, which suggested that the transcription of mitochondrial genes could be initated by primers containing polyAs. This finding explained why all of the TISs resided in A-enriched regions. However, further studies are necessary to support these explanations.

### Be Careful With Design of Experiments on ncRNAs

As it has been accepted that yeast and human cells transcribe almost their entire genomes, a huge mass of hidden or cryptic ncRNAs, particularly lncRNAs, has been identified (Houseley and Tollervey, 2009). However, some of them are basic transcriptional noise (Houseley and Tollervey, 2009), fragments from RNA degradation or random breaks during experimental processing. The correct identification of lncRNAs, particularly steady lncRNAs, has not been addressed before this study. Using the incomplete annotation of genome, researchers could misinterpret the results from experiments on ncRNAs. Here is a typical example. In a previous study, Lee et al. (2009) designed RNAi experiments to show that the knockdown of tRF-1001 impaired cell proliferation. However, tRF-1001 belongs to 5′ sRNAs from lncTRS-TGA1-1, which is an antisense gene of HERC4 (Figure 1B). Therefore, the knockdown experiments using siRNA duplexes in that study could result in the decrease in the expression of both lncTRS-TGA1-1 and HERC4. We suggest to use single-stranded siRNAs, instead of siRNA duplexes, to knockdown these 5′ sRNAs and then compare the results to those using over-expression of HERC4, since 5′ sRNAs from lncTRS-TGA1-1 could inhibit the expression of HERC4 via RNAi or similar mechanisms based on new findings in this study. We also found that most of human tRNA genes have downstream lncRNA genes as lncTRS-TGA1-1 and the 5′ sRNAs of these lncRNAs could perform molecular functions by inhibiting the expression of their antisense genes.

### Analysis of RNA Degradation Using 5′, 3′ and Intronic sRNAs

**FIGURE 4:**
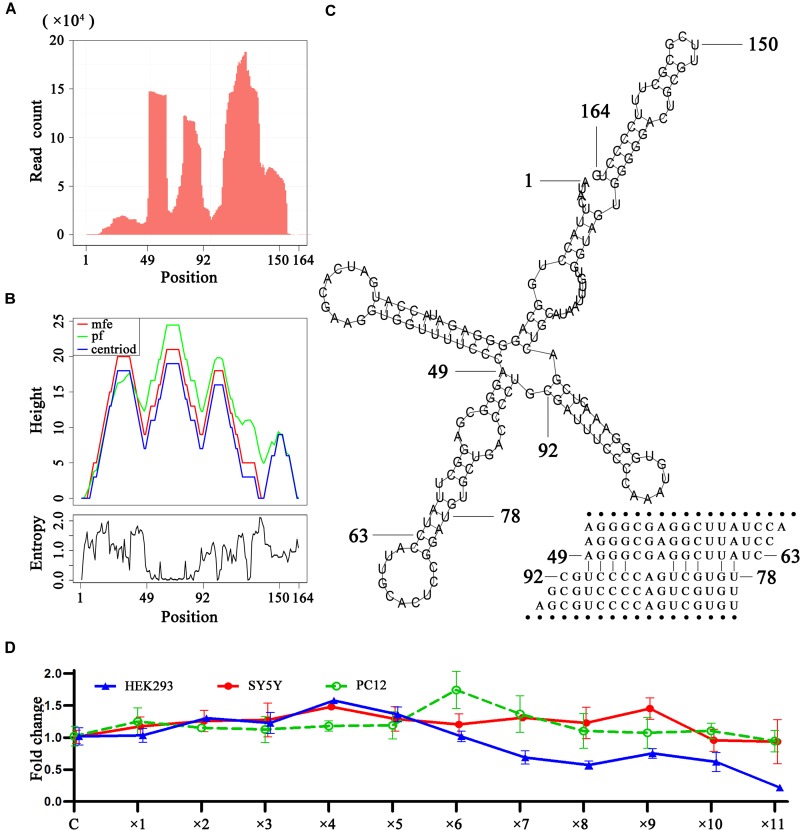
SiRNA duplexes discovered from U1 snRNAs. **(A)** The count distribution of all aligned reads on the reference U1 snRNA (RefSeq: NR_004430.2). **(B)** The above is a mountain plot representation of the MFE structure, the thermodynamic ensemble of RNA structures and the centroid structure. The positional entropy for each position is showed below. **(C)** The secondary structure of U1 snRNA. **(D)** U1 over-expression in the HEK293 (human), SY5Y (human) and PC-12 (rat) cell lines were conducted by virus transfection. The qPCR results showed the relative expression levels of U1 in 12 groups. For each experiment, 12 groups of samples named control, ×1, ×2, ×3, ×4, ×5, ×6, ×7, ×8, ×9, ×10, and ×11 were transfected by 0, 1, 2, 3, 4, 5, 6, 7, 8, 9, 10, and 11 μL U1-packaged lentiviruses The control group used unprocessed samples.

As 5′, 3′ and intronic sRNAs are degraded fragments of mature RNAs, they can be used to investigate RNA degradation (Houseley and Tollervey, 2009), particularly that of steady RNAs. The analysis of sRNA-seq data showed that in general, 5′ and 3′ sRNAs were more abundant than intronic sRNAs and short 5′ and 3′ sRNAs were more abundant than longer ones for tRNAs, rRNAs, snRNAs and mitochondrial RNAs. This suggested that these mature RNAs, particularly short RNAs (e.g., tRNAs), were mainly degraded by 3′ and 5′ exonucleases to accumulate 5′ and 3′ sRNAs. As for rRNAs and snRNAs, we found many peaks representing intronic sRNAs in the body of genes, which were significantly higher than the peaks representing 5′ or 3′ sRNAs in the genome-alignment map. In addition, the peaks representing intronic sRNAs in rRNAs showed tissue specificities. Liver tissue (SRA: SRP002272) exhibited specific peaks at position 12,891. Plasma (SRA: SRP034590) exhibited specific peaks at positions 5,431, 9,891, and 11,158. B-cells and exosome (SRA: SRP046046) exhibited specific peaks at positions 3,789 and 9,891. Platelets (SRA: SRP048290) exhibited specific peaks at positions 4,384 and 10,627. A more comprehensive study of these tissue specificities was beyond the scope of this study. Instead, we focused on a study of the secondary structures around these peaks in rRNAs and snRNAs and found that some of them were involved in dsRNA regions. In particular, we found a featured peak spanning a 43-bp region from 49 bp to 92 bp of U1 snRNA (Figure 4). In this region, the 5′ ends of most intronic sRNAs were precisely aligned to 49 or 78 bp (Figure 4A). We also found a series of duplexes with lengths from 15 bp to at least 25 bp (Figure 4C) from the 43-bp region forming a hairpin in the secondary structure of U1 snRNA. The most abundant reads AGGGCGAGGCTTATC and TGTGCTGACCCCTGC formed a 15-bp duplex structure. The second most abundant reads AGGGCGAGGCT and TGTGCTGACCC formed a 11-bp duplex structure. 99.97% (49,889/49,903) of these duplexes were found from 14 samples of plasma (SRA: SRP034590) and the duplex ratio of AGGGCGAGGCTTATC against TGTGCTGACCCCTGC was 2.15 (34,065/15,824) and, which suggested that this dsRNA region was cleaved by the RNase III family (Nicholson, 2013) to produce these siRNA duplexes (Niu et al., 2017) and could induce RNAi. This 15-bp and 11-bp duplex structures from U1 snRNA are symmetric with 2-bp overhangs at the 5′ and 3′ ends, while duplexes from other snRNAs are not. For example, the most abundant reads AAAATTGGAACGATACAGAGAA and TGAAGCGTTCCATATTTTT from U6 snRNA formed a asymmetric duplex structure, which still suggested that this dsRNA region was cleaved by the RNase III family and could induce RNAi. Based on the findings in this study, we hypothesize that 5′ and 3′ exonucleases are more prevalent than endonucleases for the degradation of mature non-coding RNAs, hence the abundance of 5′ and 3′ sRNAs observed using sRNA-seq data. The longer mature RNAs have more and longer dsRNA regions (e.g., 15 bp long for stems in U1 snRNAs) than shorter ones (e.g., 7–9 bp as the longest for stems in tRNAs) to induce dsRNA cleavage to produce siRNA duplexes. Although the vast majority of the lengths of siRNA duplexes revealed in this study were concentrated at 15 bp (section “Conclusion and Discussion”), we still hypothesized that they could induce RNAi due to the unbalanced duplex ratio of 2.15. As RNAi regulates the expression of these genes through a negative feed-back mechanism, we designed preliminary experiments to over-express U1 snRNA in the HEK293 (human), SY5Y (human), and PC-12 (rat) cell lines to prove our hypothesis. The basic idea was that if the negative feed-back mechanism existed, the expression level of U1 snRNA would decrease rather than remain stable once its over-expression surpassed a threshold. The experimental results showed that the expression level of U1 snRNA decreased after ×4, ×9, and ×6 dosages (section “Materials and Methods”) in the HEK293 (human), SY5Y (human), and PC-12 (rat) cell lines, respectively (Figure 4D). In particular, the results in the HEK293 cell line showed a significant effect caused by the negative feed-back mechanism. Therefore, RNAi could be involved in the RNA degradation and regulation of gene expression in U1 snRNA.

## Conclusion and Discussion

In this study, we used pan RNA-seq analysis to reveal the ubiquitous existence of both 5′ and 3′ end small RNAs. 5′ and 3′ sRNAs alone can be used to annotate nuclear non-coding and mitochondrial genes at 1-bp resolution and identify new steady RNAs. The identification of new steady RNAs lead to the discovery of new genes (e.g., MDL1 and MDL1AS), new biological functions and even new mechanisms. In our previous study on human rRNA genes (Chen et al., 2017), we hypothesized that 5′ and 3′ sRNAs performed biological functions and they are likely to have detrimental effects on the regulation of gene expression, as RNA degradation intermediates (Houseley and Tollervey, 2009). Cellular experiments showed the RNAi knockdown of one 20-nt degraded fragment “ATTCGTAGACGACCTGCTTC” from 28S rRNA induced cell apoptosis and inhibited cell proliferation (Chen et al., 2017). Additional investigation of the biological functions of 5′ and 3′ sRNAs was beyond the scope of this study.

Using 5′, 3′ and intronic sRNAs, we reported for the first time that enzymatic dsRNA cleavage and RNAi might be involved in the RNA degradation and gene expression regulation of U1 snRNA in humans. The function of RNAi in RNA degradation was reported as an inappropriate event in yeast rRNA and tRNA degradation and only happened when 5′ and 3′ degradation were absent (Buhler et al., 2008). However, our findings suggest that the function of RNAi in RNA degradation might be a general mechanism. Based on a previous study, the Rnt1p protein cleaves hairpin structures in pre-rRNAs, pre-mRNAs and transcripts containing non-coding RNAs (e.g., snoRNAs) for their maturation in yeast. Rnt1p recognizes the tetraloops [A/u]GNN and cleaves the stems ∼14–16 bp from the hairpin structures (Nicholson, 2013). The most abundant read AGGGCGAGGCTTATC discovered in this study contained AGGG and AGGC tetraloops and had a length of 15 bp. This suggested that Rnt1p-like enzymes could produce siRNA duplexes from U1 snRNAs but Rnt1p has yet to be reported in human to the best of our knowledge. This finding also contradicted our basic knowledge that Dicer is required for RNAi in mammal, producing siRNA duplexes with lengths of ∼20–25 bp. As members of RNase III family, both Rnt1p and Dicer have RIIIDa, RIIIDb, and dsRBD domains. Rnt1p in *Saccharomyces cerevisiae* contains a 155-aa N-terminal domain (NTD), whereas Dicer and Drosha in human have much longer N-terminal. The structure of Rnt1p post-cleavage complex shows that a novel RNA-binding motif (RBM) recognizes the guanine nucleotide in the [A/u]GNN tetraloop and that NTD and dsRBD function as two rulers measuring the distance between the tetraloop and the cleavage site (Song et al., 2017). Although our preliminary experiments supported the existence of RNAi, the identity of the enzyme that caused 15-bp duplexes in U1 snRNAs remains unclear.

The ancestral function of RNAi is generally agreed to have been immune defense against exogenous genetic elements such as transposons and viral genomes (Buchon and Vaury, 2006). However, our findings have rediscovered dsRNA cleavage and RNAi. Our rediscovery is that both dsRNA cleavage and RNAi are housekeeping systems rather than immune defense systems. Basically, enzymatic dsRNA cleavage is responsible for RNA processing, maturation and degradation, while RNAi regulates gene expression via highly efficient RNA degradation. RNAi of one gene produces siRNA duplexes that regulate expression levels of itself or other genes. Mature RNAs containing a greater number of hairpin structures have more chances to induce RNAi, which is important for highly expressed genes (e.g., U1 snRNA) or viral genes. As DNA complemented palindromes are prone to produce dsRNA regions, viruses containing a greater number of such DNA complemented palindromes in their genomes have more chances to induce RNAi for the regulation of gene expression, which is important for their infectivity and pathogenesis. In addition, we reported for the first time the existence of complemented palindromic small RNAs (RNAs) and proved that one cpsRNA from a 22-bp DNA complemented palindrome in the SARS-CoV genome could induced RNAi (Liu C. et al., 2018).

We provided a different perspective on the regulation of gene expression in U1 snRNA. The primary function of U1 snRNA is its involvement in the splicing of pre-mRNAs in nuclei. In the past 20 years, research on U1 snRNA has focused on its primary function, particularly as it relates to neurodegenerative diseases caused by abnormalities in U1 snRNA (Cheng et al., 2017b). In one of our previous studies, we reported that over-expression of U1 snRNA induced a decrease in U1 spliceosome function associated with Alzheimer’s disease. However, the relationship between U1 snRNA over-expression and U1 snRNP loss of function remains unknown (Cheng et al., 2017a). In another study, we reported that U1 snRNA over-expression induced cell apoptosis in SY5Y cells, but not in PC-12 cells (Cheng et al., 2017b). This inconsistent result can be explained by considering the function of RNAi in the RNA degradation of U1 snRNA. Though SY5Y cells and PC-12 cells exhibited different responses to U1 snRNA over-expression, both of them displayed phenomena caused by the negative feedback mechanism (Figure 4D). Using the human931 sRNA-seq dataset (section “Materials and Methods”), we also found that sRNAs of U1 snRNA were enriched in brain (SRA: SRP021924) but only a few of them were siRNA duplexes. It suggested that RNAi did not take a major role in the degradation of U1 snRNA in brain. This finding helped better understanding of neurodegenerative diseases caused by abnormalities in U1 snRNA.

We also provided a novel view on cancer and virus-induced diseases. In one of our previous studies, we reported that U1 snRNA over-expression affected the expression of mammal genes on a genome-wide scale and that U1 snRNA could regulate cancer gene expression. This was explained by the fact that alternative splicing (AS) and alternative polyadenylation (APA) were deregulated and exploited by cancer cells to promote their growth and survival (Spraggon and Cartegni, 2013). Our alternate explanation is that the over-expressed U1 snRNA in cancer cells recruits excess RNase III for RNAi, thereby causing RNase III to lose its abilities to function in the RNA degradation of other genes or in genome surveillance (Nicholson, 2013). Viruses also recruit excess RNase III, prompting RNase III to lose its abilities to function in host defense as well as its regular functions.

## Materials and Methods

### Datasets and Data Analysis

Data in four projects (SRP002272, SRP034590, SRP046046, and SRP048290) were selected from the human931 sRNA-seq dataset to build one sRNA-seq dataset for this study. Human931 was built using 931 runs of human sRNA-seq data downloaded from the NCBI SRA database (Wang et al., 2016). 15, 14, 12, and 6 runs of sRNA-seq data in these four projects were produced using Illumina sequencing technologies with length 35∼46, 101, 101, and 101 bp, respectively. One CAGE-seq dataset, one GRO-seq dataset (Bouvy-Liivrand et al., 2017) and one PacBio cDNA-seq dataset (Gao et al., 2017) were used to validate the annotations. The cleaning and quality control of sRNA-seq, CAGE-seq and GRO-seq data were performed using the pipeline Fastq_clean (Zhang et al., 2014) that was optimized to clean the raw reads from Illumina platforms. To simply annotate genes from a sequenced genome, we aligned all the cleaned reads from sRNA-seq, CAGE-seq, and GRO-seq data to the reference sequences using the software bowtie v0.12.7 allowing one mismatch. Then, we obtained SAM, BAM, sorted BAM, Pileup files using the software samtools (Zhang et al., 2016). One perl script (Supplementary Table S1) was used to transform Pileup files into 5-end files. Statistical computation and plotting were performed using the software R v2.15.3 with the Bioconductor packages (Gao et al., 2014).

### Validation by Preliminary Experiments

U1 over-expression in the HEK293 (human), SY5Y (human), and PC-12 (rat) cell lines were conducted by virus transfection using the pLVX-shRNA1 plasmids and the Lenti-X HTX Packaging System (Clontech, United States), which had been described in our previous study (Cheng et al., 2017a). U1 snRNAs of human and rat used synthetic DNA containing the sequence (RefSeq: NR_004430.2) and the sequence (GenBank: V01266.1), respectively. For each experiment, 12 groups of samples named control, ×1, ×2, ×3, ×4, ×5, ×6, ×7, ×8, ×9, ×10, and ×11 were transfected by 0, 1, 2, 3, 4, 5, 6, 7, 8, 9, 10, and 11 μL U1-packaged lentiviruses (Figure 4D). Each group contained three samples for biological replicates and the control samples used unprocessed cells. Each sample contained 10^5^ cells and virus titer was 10^7^ TU/mL for 1X. After transfection, RNA extraction, cDNA synthesis and cDNA amplification were performed following the same procedure in our previous study (Cheng et al., 2017b). For each sample, total RNA was isolated using RNAiso Plus Reagent (TaKaRa, Japan) and the cDNA was synthesized by Mir-X miRNA First-Strand Synthesis Kit (Clontech, United States). The cDNA product was amplified by qPCR (Thermo Fisher Scientific, United States) using U6 snRNA as internal control under gene-specific reaction conditions. U1 snRNAs of human and rat used the forward and reverse primers GGGAGATACCATGATCAC and CCACTACCACAAATTATGC. U6 snRNAs of human and rat used CGGCAGCACATATACTAA and GAACGCTTCACGAATTTG. The qPCR reaction mixture was incubated at 95°C for 30 s, followed by 40 PCR cycles (5 s at 95°C, 5 s at 60°C, and 10 s at 68°C for each cycle) using Hieff qPCR SYBR Green Master Mix (Yeasen, China).

## Data Availability

All NGS data and reference sequences are available in the NCBI SRA or RefSeq databases. Their accession numbers are provided in the method section.

## Author Contributions

SG conceived the project and drafted the main manuscript. SG and ZeC supervised this project. SG, HJ, and XJ analyzed the data. XY, YL, and TZ curated the sequences and prepared all the figures, tables, and additional files. XX, ZhC, and QZ performed the experiments. JR and WB revised the manuscript. All authors have read and approved the manuscript.

## Conflict of Interest Statement

The authors declare that the research was conducted in the absence of any commercial or financial relationships that could be construed as a potential conflict of interest.

## Supplementary Material

TABLE S1 All tRNA and snRNA sequences.
